# Neck circumference as an anthropometric marker of overweight among school-aged children in Bharatpur, Nepal: A community-based study

**DOI:** 10.1371/journal.pone.0354997

**Published:** 2026-07-29

**Authors:** Subina Bajracharya, Sadikshya Neupane, Parita Shrestha

**Affiliations:** 1 Department of Pediatric Nursing, School of Nursing, Chitwan Medical College, Bharatpur, Nepal; 2 Department of Community Health Nursing, School of Nursing, Chitwan Medical College, Bharatpur, Nepal; University of Montenegro-Faculty of Medicine, MONTENEGRO

## Abstract

**Background:**

Childhood overweight have become a major public health concern among children in developing countries. BMI, commonly used to diagnose overweight, requires standardized equipment and trained personnel, limiting their feasibility in large-scale screenings. Neck circumference (NC) has emerged as a simple and practical alternative for identifying overweight in children. This study aimed to examine the association between NC and established anthropometric indicators and to determine optimal NC cutoff values for identifying overweight among school-aged children.

**Methods:**

A community-based descriptive cross-sectional study was conducted from July to October 2025 among 300 apparently healthy children aged 6–11 years in Ward No. 16, Bharatpur Metropolitan City, Nepal. Sociodemographic data were collected using structured interviews. Anthropometric measurements including height, weight, NC, waist circumference (WC), and hip circumference (HC) were obtained using standardized procedures. BMI-for-age classifications were based on WHO AnthroPlus growth references. Chi square test, pearson correlation, logistic regression, and receiver operating characteristic (ROC) curve analyses were performed using SPSS version 16. Statistical significance was set at p < 0.05.

**Results:**

Among the 300 children, 48.3% had normal BMI-for-age, 10.3% were overweight and 41.3% were underweight. Chi square test revealed a statistically significant association between neck circumference and BMI-for-age (p < 0.001). Pearson correlation analysis demonstrated strong positive correlations between NC and weight (r = 0.792), height (r = 0.639), BMI (r = 0.723), waist circumference (r = 0.797), and hip circumference (r = 0.734) (p < 0.001 for all). ROC curve analysis indicated excellent diagnostic accuracy of NC for identifying overweight. The optimal cutoff values were 27.75 cm for boys (AUC = 0.884; sensitivity = 88.9%; specificity = 66.2%) and 26.75 cm for girls (AUC = 0.904; sensitivity = 100%; specificity = 69.4%). The overall cutoff value was 27.25 cm (AUC = 0.883; sensitivity = 80.6%; specificity = 74.3%). Multivariable logistic regression analysis confirmed NC had a statistically significant positive association with overweight with COR 12.077 (4.75–30.67) and AOR 20.817 (6.34–68.29).

**Conclusions:**

Neck circumference is significantly correlated with established anthropometric indicators and demonstrates diagnostic accuracy for identifying overweight among school-aged children. NC measurement is a simple, reliable, and cost-effective screening tool that may be particularly useful in school-based and resource-limited settings.

## 1. Introduction

Malnutrition, encompassing both undernutrition and overweight (overnutrition), remains a pressing public health issue among children, particularly in developing countries like Nepal, despite ongoing global efforts to improve child nutrition. Nepal is currently experiencing a nutritional transition characterized by the coexistence of undernutrition and rising overweight, a phenomenon known as the double burden of malnutrition. Though Nepal’s 2015 Constitution establishes food sovereignty as a fundamental right [1], over one-third of children under five still suffer from malnutrition [2]. Yet different studies show the prevalence of overweight among school children ranged from 10% to 18% [3,4].

Overweight among school-age children have emerged as major global public health concerns over the past few decades. Once considered problems limited to high-income countries, childhood overweight is now rapidly increasing in low-and middle-income nations, including Nepal. Childhood overweight frequently tracks into adolescence and adulthood, significantly increasing the lifetime risk of cardiovascular disease and other non-communicable diseases [5,6].

While undernutrition remains prevalent, increased availability of processed foods, urban lifestyle patterns, and reduced outdoor activity have contributed to a growing proportion of overweight children, particularly in urban and semi-urban settings. This dual burden presents a complex public health challenge, requiring efficient, accessible, and context-appropriate screening strategies [7].

Nutritional status is a critical health indicator for school age children experiencing rapid physical and cognitive development. Early identification of overweight in school-age children is essential for timely intervention and prevention of long-term complications. While traditional anthropometric indicators like BMI and weight/height ratios are commonly used for nutritional assessment, they present challenges including measurement inconsistencies and requirements for trained personnel and specialized equipment. Neck circumference (NC) has emerged as a promising alternative that reflects upper body subcutaneous fat distribution, that is less influenced by daily fluctuations than conventional measures and has demonstrated associations with metabolic risks factors making it a convenient and reliable tool for identifying overweight in children [8,9].

Although pediatric NC research began in 2010 and international studies have demonstrated its potential for identifying overweight, obesity, and metabolic syndrome, widespread clinical adoption remains limited due to the absence of universally accepted cut-off values for children [10]. Therefore, this study aimed to examine the association between NC and established nutritional indicators, and determine appropriate NC cutoff values for identifying overweight, ultimately contributing to the development of a simple, cost-effective approach to early nutritional screening in children, especially for the resource limited setting like Nepal.

## 2. Materials and methods

### 2.1. Study design, setting and population

A community-based, descriptive cross-sectional study was conducted from July to October 2025 at ward no. 16, a semi-urban setting in Bharatpur Metropolitan City, Chitwan District, Nepal. The study population comprised apparently healthy school-age children aged 6–11 years, primarily pre-pubertal, residing within the designated ward boundaries whose parents or legal guardians provided written informed consent.

Considering the prevalence of 25.7% [10], level of significance at 95% and allowable error at 5% sample size was calculated using the formula n = Z^2^ pq/d^2^. A total of 300 children were selected as sample for this study. Participants were recruited through non-probability consecutive sampling technique.

### 2.2. Data collection

Sociodemographic information was obtained through face-to-face interviews using a pre-tested, structured questionnaire administered to parents or guardians. Anthropometric data of children, including height, weight, neck circumference, waist circumference, and hip circumference were recorded by a single investigator eliminating inter-observer bias. Data were collected using weighing machine, stadiometer and flexible measuring tape. For height measurement, each participant was made to stand barefoot against the rod of stadiometer with heels, buttocks, and shoulders touching it, head held in Frankfurt horizontal plane and marked at the level of the occipital region with the head piece to the nearest 0.1 cm. For weight measurement, pre-calibrated electronic weighing scale was used. The machine was checked for a zero error. Weight was measured without shoes or extra clothing, to the nearest 0.1 kg.

NC was measured between the mid cervical spine and mid anterior neck, using a flexible measuring tape to the nearest 0.1 cm with the child in the standing position, head held erect and eyes facing forwards and neck in the horizontal plane at the level of most prominent position, the thyroid cartilage. WC was measured by using flexible measuring tape to the nearest 0.1 cm with the child standing, and at the end of normal expiration at a point midway between the inferior margin of the lowest rib and the iliac crest. HC was measured at the maximum circumference around the buttocks. All measurements were taken twice and the average of two readings was used for the analysis. To maintain intra observer reliability same standardized protocol, same anatomical landmark, same position, and the same measuring tape was used each time for all the measurements. Each session included a study explanation and required approximately 20–25 minutes per participant.

### 2.3. Data analysis

Waist-To-Hip Ratio (WHR) was calculated by dividing WC by HC. BMI kg/m^2^ was calculated using WHO Anthroplus software and interpreted according to WHO guidelines. BMI for age and sex percentile growth curves were used to classify the children and was defined as underweight (<−2 SD), normal weight (Between −2 SD and +1 SD), and overweight (> +1 SD) [11].

For the statistical analysis, Statistical Package for the Social Sciences (SPSS) version 16 was used. Data were expressed in terms of mean and standard deviation. Chi square test was used to measure the association between NC and BMI for age. Pearson correlation coefficient was applied to test correlation between the NC and other variables like age, height, weight, BMI, WC, HC and WHR. Receiver operating characteristic (ROC) curve analysis was used to find out the ability of NC to identify correctly children with high BMI, and to determine the best NC cut-off point for identifying children as overweight. A test with an area under the curve (AUC) 0.85 is considered an accurate test [12]. The best cutoff values were established for male and female children separately. Statistical significance was set at p < 0.05 for all analyses.

### 2.4. Ethics statement

Ethical approval was obtained from the Institutional Review Committee (IRC) of Chitwan Medical College (Approval No.: CMC-IRC/081/082–115; Date: June 8, 2025), Bharatpur, Chitwan before commencing the study. Written informed consent was obtained from parents or legal guardians of all participants after explaining the study objectives, procedures, potential risks, and benefits in the local language (Nepali). Assent was obtained from children. Participation was entirely voluntary, and participants were informed of their right to withdraw from the study at any time without penalty or loss of benefits. Confidentiality was maintained throughout the study by assigning unique identification codes.

## 3. Results

A total of 300 children aged 6–11 years were included in the study. The largest age group was 11 years (19.0%), followed by 6 and 8 years (18.3% each). More than half of the participants were male (54.3%). The majority belonged to the Brahmin/Chhetri ethnic group (50.3%), and the highest proportion were studying in Grade 1 (27.0%) (Table 1). The age specific and sex specific mean ± standard deviation of age, height, weight, BMI, neck circumference (NC), waist circumference (WC), hip circumference (HC), and waist-hip ratio (WHR) are presented in Table 2 and 3.

**Table 1:** Socio-demographic characteristics of children n = 300.

Variables	Number	Percent
**Age in years**		
6	55	18.3
7	51	17.0
8	55	18.3
9	38	12.7
10	44	14.7
11	57	19.0
**Sex**		
Male	163	54.3
Female	137	45.7
**Ethnicity**		
Brahmin/Chhetri	151	50.3
Janajati	82	27.3
Dalit	39	13.0
Madhesi	28	9.3
**Grade**		
1	81	27.0
2	43	14.3
3	69	23.0
4	33	11.0
5	33	11.0
6	26	8.7
7	15	5.0

**Table 2:** Age specific anthropometric characteristics of children n = 300.

Age in years	6	7	8	9	10	11
**n**	55 (18.3%)	51 (17%)	55 (18.3%)	38 (12.7%)	44 (14.7%)	57 (19%)
**Height (cm)**	113.73 ± 5.21	122.39 ± 5.41	127.07 ± 5.80	132.92 ± 6.66	137.48 ± 6.59	145.44 ± 10.44
**Weight (kg)**	18.57 ± 2.16	21.80 ± 3.57	24.81 ± 5.17	28.11 ± 5.69	30.04 ± 4.85	36.48 ± 8.78
**BMI (kg/m**^**2**^)	14.36 ± 1.41	14.52 ± 1.76	15.26 ± 2.16	15.85 ± 2.72	15.79 ± 1.85	17.09 ± 2.91
**NC (cm)**	25.38 ± 1.23	25.69 ± 1.65	26.54 ± 1.69	27.32 ± 2.07	27.13 ± 1.34	28.64 ± 1.72
**WC (cm)**	51.39 ± 3.09	52.75 ± 4.49	54.43 ± 5.02	57.92 ± 7.38	58.72 ± 5.64	62.08 ± 7.29
**HC (cm)**	58.89 ± 4.09	62.09 ± 4.27	65.37 ± 6.15	67.97 ± 6.98	70.06 ± 4.92	76.24 ± 8.39
**WHR**	0.87 ± 0.04	0.85 ± 0.05	0.83 ± 0.04	0.85 ± 0.06	0.83 ± 0.06	0.81 ± 0.06

Continuous variables are shown as Mean±Standard deviation.

**Table 3:** Sex specific anthropometric characteristics of the children n = 300.

Variables	Sex		Overall
	Male (n-163)	Female (n-137)	
Age (years)	8.45 ± 1.81	8.49 ± 1.78	8.47 ± 1.79
Height (cm)	129.14 ± 11.87	130.12 ± 13.76	129.59 ± 12.76
Weight (kg)	26.27 ± 7.48	26.91 ± 8.92	26.65 ± 8.16
BMI (kg/m^2^)	15.46 ± 2.25	15.48 ± 2.52	15.47 ± 2.37
NC (cm)	27.34 ± 1.84	26.21 ± 1.96	26.77 ± 1.97
WC (cm)	56.76 ± 6.68	55.35 ± 6.79	56.11 ± 6.76
HC (cm)	65.52 ± 7.55	68.12 ± 9.07	66.71 ± 8.36
WHR	0.86 ± 0.05	0.81 ± 0.05	0.84 ± 0.05

Continuous variables are shown as Mean±Standard deviation.

**Fig 1:**
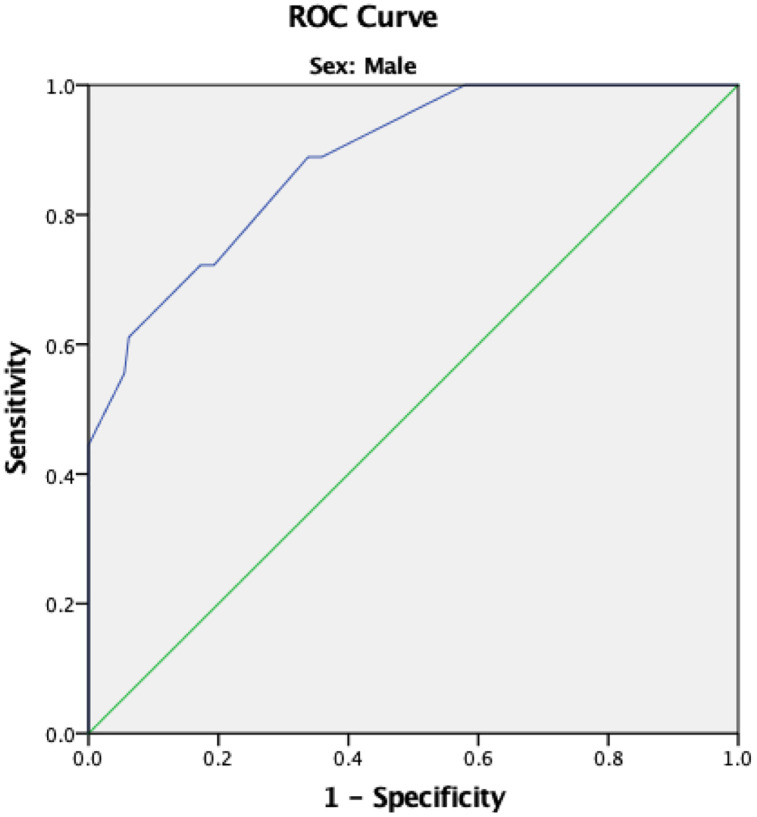
Receiver operating characteristics (ROC) curve of boys.

**Fig 2:**
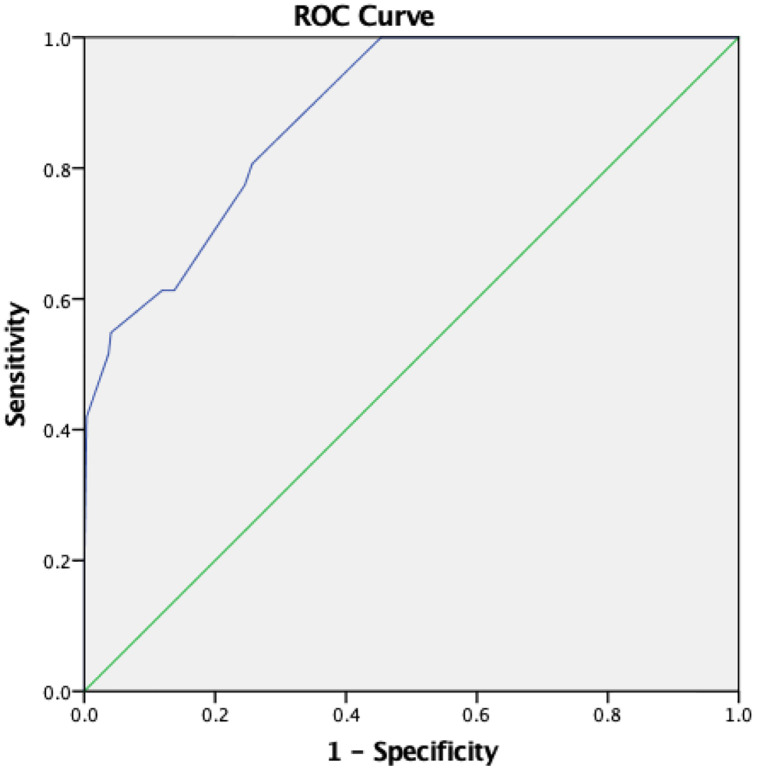
Receiver operating characteristics (ROC) curve of girls.

**Fig 3:**
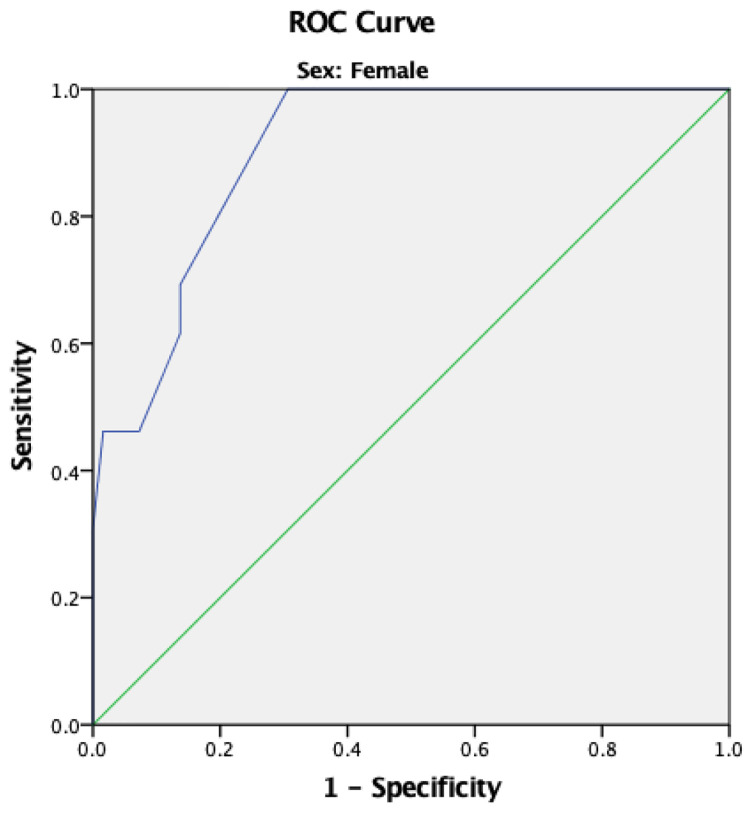
Receiver operating characteristics (ROC) curve of total sample.

Based on the Receiver operating characteristic (ROC) curve analysis, sensitivities, specificities, (Figs 1–3), the optimal NC cut-off by age and sex was identified. The cut-off for NC ranged from 26.75 cm-29.25 cm across the age group of 6–11 years. Neck circumference increased with age. In all age-groups of both genders, diagnostic performance of NC was ‘highly accurate’ in classifying overweight (AUC = 0.85 to 0.97) (Table 4). The cut-off was 27.75 cm for boys (sensitivity: 88.9%; specificity: 66.2%; AUC: 0.884) and 26.75 cm for girls (sensitivity: 100%; specificity: 69.4%; AUC: 0.904). The aggregate threshold was established at 27.25 cm, yielding 80.6% sensitivity and 74.3% specificity, with an overall AUC of 0.883 (Table 5).

**Table 4:** Cut-off point, sensitivity and specificity of neck circumference for detecting overweight in children by age.

Age (years)	NC (cm)	AUC	p-value	95% CI	Sensitivity	Specificity	Youden’s Index (J)
6	26.75	0.88	0.186	0.77-1	1	0.81	0.81
7	26.75	0.85	0.041	0.73-0.97	1	0.70	0.70
8	26.75	0.88	0.001	0.79-0.98	1	0.63	0.63
9	28.5	0.97	0.000	0.92-1	0.83	1	0.83
10	27.5	0.80	0.048	0.60-0.99	0.75	0.67	0.42
11	29.25	0.96	0.000	0.92-1	1	0.83	0.83

**Table 5:** Cut-off point, sensitivity and specificity of neck circumference for detecting overweight in children by sex.

Sex	NC (cm) Cutoff	AUC	p-value	95% CI	Sensitivity	Specificity	Youden’s Index (J)
Male	27.75	0.88	0.00	0.80-0.96	0.88	0.66	0.55
Female	26.75	0.90	0.00	0.84-0.96	1	0.69	0.69
Total sample	27.25	0.88	0.00	0.82-0.93	0.80	0.74	0.54

According to BMI-for-age classification, 145 (48.3%) children had normal BMI, 124 (41.3%) were underweight, and 31 (10.3%) were overweight, none of the children were obese Neck circumference classification based on the identified cut-off demonstrated that majority of participants (n = 206, 68.7%) fell into not overweight category (Table 6). BMI was further recategorized into 2 categories not overweight (combining underweight and normal weight), and overweight. A statistically significant association between neck circumference and BMI-for-age (p < 0.001) was observed (Table 7).

**Table 6:** Nutritional status of the children n = 300.

Variables	Number	Percent
**BMI for age**		
Underweight	124	41.3
Normal	145	48.3
Overweight	31	10.3
**Neck circumference**		
Not overweight	206	68.7
Overweight	94	31.3

**Table 7:** Association between neck circumference and BMI n = 300.

Variables	Neck Circumference		χ^2^	*p*-value
	Not overweight	Overweight		
	No. (%)	No. (%)		
**BMI for age**			39.073	0.00
Not overweight	200 (74.3%)	69 (25.7%)		
Overweight	6 (19.4%)	25 (80.6%)		

*Significant association at p < 0.05.

The correlation coefficients of NC with other anthropometric measurements are displayed in Table 8. It indicates a statistically significant positive correlation between neck circumference and height, weight, BMI, waist circumference, and hip circumference (p < 0.001 for all). In contrast, no significant correlation was observed between neck circumference and waist-hip ratio (p = 0.386).

**Table 8:** Pearson correlation between neck circumference and other anthropometric variables.

Variables	Neck Circumference	
	r	*p-*value
Height	0.639	0.000
Weight	0.792	0.000
BMI	0.723	0.000
Waist Circumference	0.797	0.000
Hip Circumference	0.734	0.000
Waist Hip Ratio	0.050	0.386

*Significant association at p < 0.05.

Table 9 displays the multivariable logistic regression analysis that confirmed NC had a statistically significant positive association with overweight. The crude ORs for overweight was 12.077 (4.75–30.67) and age and sex adjusted ORs for overweight was 20.817 (6.34–68.29). All the predictors within the multivariable logistic regression model satisfied the condition of coloniality through the variance inflation factor (VIF). Regarding the model adequacy, the overall goodness of fit is indicated by non-significant chi square value (p > 0.05). The Hosmer- Lameshow statistics has chi square value of 2.238 and a p value of 0.97, which means that the p value is not statistically significant. So, this model is quite a good fit. The pseudo R square estimates indicate approximately 12.3% or 25.2% of variation in the dependent variable.

**Table 9:** Multivariable logistic regression for predicting overweight among school age children.

	Model	Overweight OR (95% CI)	*p*-value
**Neck circumference (cm)**	**Model I**	12.077 (4.75-30.67)	0.000
	**Model II**	20.817 (6.34-68.29)	0.000

All VIF < 2, OR: odds ratio, CI: confidence interval, Model I: without adjustment, Model II: Adjusted for age and sex..

Hosmer & Lameshow test: _χ_^2^ 2.238, p-0.97, Pseudo R Square (Cox & Snell R square: 0.123, Nagelkerke R square: 0.252).

## 4. Discussion

NC is increasingly recognized as a practical anthropometric indicator for identifying overweight in childhood. However, its application in pediatric populations remains limited due to the lack of standardized reference values. The present study examined the utility of NC for detecting overweight among school-aged children (6–11 years) and established sex-specific cutoff points with good diagnostic performance.

### 4.1. Nutritional profile of the study population

The study revealed a dual burden of malnutrition among the children. While 48.3% of children had normal BMI-for-age, a substantial proportion were underweight (41.3%), and 10.3% were overweight. These findings reflect the coexistence of undernutrition and emerging overweight within the same population, a phenomenon increasingly observed in developing countries undergoing nutritional transition. The relatively lower proportion of overweight children compared to underweight children indicates that undernutrition remains a significant public health concern; however, the presence of overweight cases highlights the growing importance of early obesity screening.

More than 390 million children and adolescents aged 5–19 years were overweight in 2022, with a global prevalence of approximately 20% [13]. Similar findings were revealed in the study by Aguilar Liendo et. al., which showed more than a third of schoolchildren had malnutrition by excess (24% overweight and 10% obesity) [9]. Although the proportion of overweight children in the present study (10.3%) is lower than the global estimate, the presence of excess weight alongside high undernutrition reflects nutritional transition and underscores the importance of early screening strategies.

### 4.2. Association between neck circumference and anthropometric indicators

The present study demonstrated a statistically significant association between NC and BMI-for-age (p < 0.001). Pearson correlation analysis further revealed strong positive correlations between NC and weight (r = 0.792), height (r = 0.639), BMI (r = 0.723), waist circumference (r = 0.797), and hip circumference (r = 0.734), all statistically significant. However, no significant correlation was found between NC and WHR (r = 0.050, p = 0.386). ROC curve analysis in the present study demonstrated excellent discriminatory ability of NC for detecting overweight. Age specific NC cutoff ranged from 26.75 cm to 29.25 cm in different age groups with (AUC = 0.85 to 0.97). The AUC was 0.884 in boys and 0.904 in girls, with an overall AUC of 0.883, indicating high diagnostic accuracy.

These findings are consistent with previous literature demonstrating the utility of NC as an anthropometric marker of overweight in pediatric populations. A study conducted among schoolchildren aged 10–12 years reported strong correlations between NC and waist circumference as well as BMI-z (r > 0.8; p < 0.001), with AUC values exceeding 0.90 across age and sex groups (9). Similarly, Malini et al. found significant positive correlations between NC and BMI (r = 0.84 in boys and r = 0.75 in girls) and between NC and WC (r = 0.87 in boys and r = 0.84 in girls) among children under 12 years [14]. A study by Patnaik et al reveled that BMI was positively correlated with NC (r = 0.642 for boys, 0.615 for girls) and waist circumference (r = 0.693 for boys, 0.682 for girls) at significant level (p < 0.001) [15].

Furthermore, previous studies have demonstrated that NC performs relatively well in classifying overweight (AUC: 0.67–0.75, p < 0.001), general obesity (AUC: 0.81–0.85, p < 0.001), and abdominal obesity (AUC: 0.73–0.78, p < 0.001) across age groups and sexes [16]. Other investigations have similarly reported satisfactory predictive ability of NC for identifying overweight among school children [17–19]. Collectively, these findings, together with the results of the present study, reinforce the utility of NC as a practical and reliable marker for defining overweight in children.

The absence of correlation between NC and WHR in the current study may be explained by limited variability in WHR among children and developmental differences in fat distribution during prepubertal years.

### 4.3. Diagnostic performance and cut-off values of neck circumference

In the current study the identified cutoff values for NC were; 27.75 cm for boys (Sensitivity 88.9%, Specificity 66.2%), 26.75 cm for girls (Sensitivity 100%, Specificity 69.4%), and 27.25 cm overall (Sensitivity 80.6%, Specificity 74.3%). These values are comparable with those reported in other studies. Asif et al. reported prepubertal NC cutoffs ranging from 26.36–26.78 cm in boys and 25.02–25.27 cm in girls, while broader age-group cutoffs ranged between 25.27–30.35 cm in boys and 25.00–31.62 cm in girls [20]. The cutoff values identified in the present study fall within these reported ranges.

Malini et al. identified a cutoff of 26.5 cm for both boys and girls aged 6–11 years, with AUC values of 0.86 in boys and 0.82 in girls; slightly lower than those observed in the present study [14]. H. T. et al. reported higher cutoff values (32 cm in boys and 30 cm in girls) with high sensitivity and specificity [21]. Variations in cutoff values across studies may be attributed to differences in ethnicity, nutritional status, age distribution, pubertal stage, and body composition. Importantly, Asif et al. also highlighted that NC cut-offs differ between prepubertal and pubertal children, suggesting that age and sex specific reference values are necessary [20]. This study, focusing on children aged 6–11 years (primarily prepubertal), identified cut-offs that are consistent with prepubertal ranges reported in the literature.

Multivariable logistic regression analysis showed NC had a statistically significant positive association with overweight indicating that children with higher NC were significantly more likely to be overweight compared to those with lower NC. The crude ORs for overweight was 12.077 (4.75–30.67) and adjusted ORs for overweight was 20.817 (6.34–68.29). After adjustment for other covariates (age and sex), children with higher NC had approximately 21-fold increased odds of overweight, supporting NC as a strong anthropometric marker of overweight status.

### 4.4. Limitations

This study has several limitations. First, Participants were recruited from a single semi-urban site using non-probability sampling, which restricts external validity and may limit the generalizability of these findings to other populations. Second, the cross-sectional design precludes inference of causal relationships between neck circumference (NC) and overweight. Third, there is no universally accepted age- and sex-specific reference standard for NC; although this study proposes locally derived cutoff values, these require further validation. Fourth, the adjusted odds ratio indicated a strong association, but the 95% confidence interval was very wide, reflecting low precision and considerable uncertainty regarding the true magnitude of the effect. Larger, multicenter studies are warranted to establish standardized NC reference charts, confirm their clinical utility, and yield more precise estimates. Fifth, the relatively small number of participants in the overweight category may have inflated sensitivity estimates and reduced the precision and generalizability of the diagnostic accuracy measures. Finally, factors such as pubertal stage, dietary habits, physical activity, parental BMI, and socioeconomic status may have influenced both NC and overweight status.

### 4.5. Implications

Neck circumference (NC) measurement offers several practical advantages that support its use in routine screening. It is a simple, rapid, and low-cost anthropometric measure that can be performed with minimal equipment and training. In addition, NC can be assessed without requiring removal of clothing, which may enhance acceptability and feasibility in community and school-based settings. Unlike waist circumference, NC is less affected by abdominal distension and recent food intake, potentially providing more stable measurements. Given its strong correlation with established anthropometric indices and its demonstrated diagnostic accuracy, NC may represent a particularly useful screening tool for identifying overweight individuals in school health programs and resource-limited settings.

## 5. Conclusion

Neck circumference was significantly associated with BMI, weight, height, waist circumference, and hip circumference and demonstrated high diagnostic accuracy for detecting overweight among children aged 6–11 years. On this basis, NC appears to be a feasible, reliable, and cost-effective anthropometric measure for screening childhood overweight, with particular applicability in resource-limited and school-based settings. Future research should focus on validating these findings in larger, multicenter cohorts, establishing standardized age and sex specific NC reference values, and evaluating the integration of NC measurement into routine school health programs to determine its impact on early identification and management of childhood overweight.

## Supporting information

S1 Dataset Data sheet. (XLSX)
