# Supplementary material for: Neck circumference as an anthropometric marker of overweight among school-aged children in Bharatpur, Nepal: A community-based study
Source: PLoS One. 2026 Jul 29;21(7):e0354997. doi: 10.1371/journal.pone.0354997 (PMC13419187; doi:10.1371/journal.pone.0354997)
Supplement: Attachment — Submitted filename: Response to Reviewers.docx [file pone.0354997.s003.docx]

**Response to Editor and Reviewers' Comments**

Manuscript title: Neck circumference as an anthropometric marker of overweight among school-aged children in Bharatpur, Nepal: a community-based study PONE-D-26-16450.

We sincerely thank the editor and all reviewers for their thorough evaluation of our manuscript and for the constructive comments provided. All comments have been carefully addressed, and the manuscript has been revised accordingly to enhance methodological rigor, clarity, and overall quality. For ease of review, all changes are highlighted in red/ tracked in the manuscript. Below, we provide a detailed, point-by- point response.

| **Comment No.** | **Comment** | **Response/Action** | **Section/ Page** |
| --- | --- | --- | --- |
| **General Comments** | | | |
| 1 | Please ensure that your manuscript meets PLOS ONE's style requirements, including those for file naming. | Thank you for this important comment. The manuscript has undergone thorough editing to meet the journal’s style. | Entire manuscript (Pages 1-End) |
| 2 | The ethical approval number(s) listed in the manuscript and/or submission metadata does not match the approval number on the ethical approval document you provided. Please ensure that all approval numbers are correct. | Thank you for highlighting this important issue. This has been corrected with the Approval No.: CMC-IRC/081/082-115. | 2.4 Ethics statement (Page 7) |
| 3 | Please remove any funding-related text from the manuscript and let us know how you would like to update your Funding Statement. | Thank you for this valuable comment. It has been removed from the manuscript. The statement “The authors received no specific funding for this work” has been included in the cover letter. | Declarations (Page 18) |
| 4 | In the online submission form you indicate that your data is not available for proprietary reasons and have provided a contact point for accessing this data. Please note that your current contact point is a co-author on this manuscript. According to our Data Policy, the contact point must not be an author on the manuscript and must be an institutional contact, ideally not an individual. Please revise your data statement to a non-author institutional point of contact, such as a data access or ethics committee, and send this to us via return email. Please also include contact information for the third-party organization, and please include the full citation of where the data can be found. | Thank you for this important comment. It has been changed to the data that support the findings of this study are available as supplementary file. We confirm that all underlying data have been made fully available as supplementary files in accordance with the journal requirements. | Supplementary file |
| 5 | Your ethics statement should only appear in the Methods section of your manuscript. If your ethics statement is written in any section besides the Methods, please delete it from any other section. | Thank you for this observation. Ethics statement are written in methods section only. | 2.4 Ethics statement (Page 7) |
| 6 | If the reviewer comments include a recommendation to cite specific previously published works, please review and evaluate these publications to determine whether they are relevant and should be cited. There is no requirement to cite these works unless the editor has indicated otherwise. | Thank you for this valuable comment. |  |
| **Reviewer 1** | | | |
| 1 | The primary concern relates to the study design and sampling strategy. Participants were selected using a non-probability consecutive sampling technique from a single ward of Bharatpur Metropolitan City. This approach limits representativeness and introduces selection bias. Since the sample was not randomly selected and came from one localized semi-urban area, the derived NC cutoff values may not generalize to broader pediatric populations in Nepal with different ethnic, nutritional, socioeconomic, or rural–urban backgrounds. The authors should discuss this limitation more explicitly and avoid overgeneralization of conclusions. | Thank you for this insightful comment. Participants were recruited from a single semi-urban site using non-probability sampling, which restricts external validity and may limit the generalizability of these findings to other populations. Although this study proposes locally derived cutoff values of NC, these require further validation. Larger, multicenter studies are warranted to establish standardized NC reference charts, confirm their clinical utility, and yield more precise estimates. These are discussed in the limitation section. | 4.4 Limitations (Page-16) |
| 2 | A second major issue concerns the statistical analysis of overweight prediction. Only 31 children (10.3%) were classified as overweight, yet logistic regression produced a very large adjusted odds ratio (AOR = 20.817, 95% CI: 6.34–68.29). With such a relatively small number of outcome events, regression estimates may be unstable and confidence intervals wide. The manuscript should clarify the number of variables entered into the model, assess events-per-variable adequacy, and explain whether assumptions of logistic regression were fully met. In addition, odds ratios for a continuous predictor such as NC should specify the unit increase (per 1 cm increase or categorized threshold). | Thank you for this observation. All the predictors within the multivariable logistic regression model satisfied the condition of coloniality through the variance inflation factor (VIF). The OR was calculated adjusted to the variables age and sex. Regarding the model adequacy, the overall goodness of fit is indicated by non-significant chi square value (p>0.05). The Hosmer- Lameshow statistics has chi square value of 2.238 and a p value of 0.97, which means that the p value is not statistically significant. So, this model is quite a good fit. The pseudo R square estimates indicate approximately 12.3% or 25.2% of variation in the dependent variable. The relatively small number of participants in the overweight category may have inflated sensitivity estimates and reduced the precision and generalizability of the diagnostic accuracy measures. It is included in the limitation section. | 3. Results  (Pages 12-13)  4.4 Limitations (Page-16) |
| 3. | The ROC analysis is interesting, but the manuscript should provide more detail regarding cutoff derivation and validation. The proposed NC thresholds were developed and tested using the same dataset, which may overestimate performance. No internal validation methods (bootstrapping, split-sample validation, cross-validation) or external validation cohort were used. Therefore, the reported sensitivity, specificity, and AUC values should be interpreted cautiously. Independent validation in another Nepali pediatric cohort would substantially strengthen the manuscript. | Thank you for this valuable comment. The issue raised are valid and are incorporated in the limitations. Independent validation in another Nepali pediatric cohort would definitely strengthen the findings. This is included in the conclusion. | 5. Conclusions (Page- 17) |
| 4. | Another concern is the data availability statement. The manuscript states that data are available from the corresponding author upon reasonable request. PLOS ONE generally expects the minimal underlying dataset to be publicly available unless justified restrictions apply. The authors should deposit de-identified data in a suitable repository or provide a stronger rationale for restricted access. Several potentially important confounders were not adequately addressed. Although age and sex were adjusted in one model, other factors such as pubertal stage, dietary habits, physical activity, parental BMI, socioeconomic status, and ethnicity may influence both NC and overweight status. Because children aged 6–11 years may vary in developmental stage, this should be acknowledged more clearly. | Thank you for this important comment. It has been changed to the data that support the findings of this study are available as supplementary file. We confirm that all underlying data have been made fully available as supplementary files in accordance with the journal requirements.  The suggestion has been incorporated in limitations. | Declaration (Page 18)  Supplementary file  4.4 Limitations (Page-16) |
| 5 | Use of BMI-for-age as the Only Reference Standard: The study evaluates neck circumference against BMI-for-age alone. While BMI is widely used, it does not directly measure body fat distribution or adiposity. Since NC is proposed as an alternative screening marker, comparison with additional standards such as skinfold thickness, waist-to-height ratio, or body composition methods would strengthen validity. | Thank you for this important comment. The study indicated a statistically significant positive correlation between neck circumference and height, weight, BMI, waist circumference, and hip circumference (p <0.001 for all). In contrast, no significant correlation was observed between neck circumference and waist-hip ratio (p=0.386). | 3. Results Table 8 (Page-12) |
| 6 | Very High Underweight Prevalence (41.3%): The sample had a strikingly high proportion of underweight children, much higher than overweight cases (10.3%). This unusual nutritional distribution may affect ROC-derived cutoffs and may not represent broader school-age populations. Authors should discuss whether the cutoff values are specific to this malnutrition-burdened population. | Thank you for this important comment. The cutoff values are calculated specific to the overweight children. | 3. Results (Page 9-11) |
| 7 | Cross-Sectional Design Limits Predictive Claim: The title uses “predictor,” but this is a cross-sectional study. It identifies association and classification ability, not prospective prediction of future overweight. Wording should be revised to avoid implying causal or longitudinal predictive power. | Thank you for this valuable comment. The title of the study has been revised to Neck circumference as an anthropometric marker of overweight among school-aged children in Bharatpur, Nepal: a community-based study. | (Page 1) |
| 8 | Older Statistical Software: Use of SPSS version 16 is not itself fatal, but it is outdated. Authors should confirm methods were properly implemented and report exact procedures used for ROC and regression analyses. | Thank you for this valuable comment. SPSS version 16 is freely available for use. New versions though available are not free and the use of corrupted version seemed unethical so were not used. The authors confirm that the analysis methods were implemented and reported properly. |  |
| 9 | Measurement Reliability Not Reported: Anthropometric measurements were performed by one investigator, but no intra-observer reliability, repeated measurement error, or calibration consistency data were provided. Since NC differences of less than 1 cm affect cutoffs, measurement precision is important. | Thank you for this valuable comment. All measurements were taken twice and the average of two readings was used for the analysis. To maintain intra observer reliability same standardized protocol, same anatomical landmark, same position, and the same measuring tape was used each time for all the measurements. | 2.2 Data collection (Pages 6-7) |
| **Reviewer #2** | | | |
| 1 | Thank you for your nice work. -Neck circumference is labeling more children as normal (68.7) vs BMI based normal range (48.3%). -NC is labeling more children as overweight (31.3%) vs BMI based overweight (10.3 %). Can you please provide explanation | Thank you for this valuable comment. The NC has been recategorized as overweight and not overweight. Regarding the higher percentage of overweight children using NC, it solely depends on single parameter i.e. neck circumference. It does not consider the height and weight of the children. Larger studies are required to confirm the association. This is included in limitation | 3. Results (Page 12)  4.4 Limitations (Page-16) |
| 2 | On Table 6: BMI has three categories while NC has two. Are the underweight children be classified as normal in the NC group? Please provide explanation and if it has resulted in the above discrepancy as well | Thank you for this valuable comment. The underweight children were classified as normal in the NC group previously which has been corrected. The NC has been recategorized as overweight and not overweight. | 3. Results (Page 12) |
| 3 | Please provide explanation for the relatively wide confidence interval (6.34–68.29) | Confidence interval was very wide, reflecting low precision and considerable uncertainty regarding the true magnitude of the effect. Larger, multicenter studies are warranted to establish standardized NC reference charts, confirm their clinical utility, and yield more precise estimates. | 4.4 Limitations (Page-16) |
