# Supplementary material for: Neck circumference as an anthropometric marker of overweight among school-aged children in Bharatpur, Nepal: A community-based study
Source: PLoS One. 2026 Jul 29;21(7):e0354997. doi: 10.1371/journal.pone.0354997 (PMC13419187; doi:10.1371/journal.pone.0354997)
Supplement: Peer review history

# Peer review: Decision Letter 0

**DOI:** 10.1371/journal.pone.0354997.r001

**Authors:** Aleksandra Klisic

1 May 2026

PONE-D-26-16450

Neck Circumference as a Predictor of Overweight among School Age Children in Bharatpur, Nepal: A Community Based Study

PLOS One

Dear Dr. Bajracharya,

Thank you for submitting your manuscript to PLOS ONE. After careful consideration, we feel that it has merit but does not fully meet PLOS ONE’s publication criteria as it currently stands. Therefore, we invite you to submit a revised version of the manuscript that addresses the points raised during the review process.

Please submit your revised manuscript by Jun 15 2026 11:59PM. If you will need more time than this to complete your revisions, please reply to this message or contact the journal office at plosone@plos.org. When you're ready to submit your revision, log on to https://www.editorialmanager.com/pone/ and select the 'Submissions Needing Revision' folder to locate your manuscript file.

Please include the following items when submitting your revised manuscript:

- A letter that responds to each point raised by the academic editor and reviewer(s). You should upload this letter as a separate file labeled 'Response to Reviewers'.
- A marked-up copy of your manuscript that highlights changes made to the original version. You should upload this as a separate file labeled 'Revised Manuscript with Track Changes'.
- An unmarked version of your revised paper without tracked changes. You should upload this as a separate file labeled 'Manuscript'.

If you would like to make changes to your financial disclosure, please include your updated statement in your cover letter. Guidelines for resubmitting your figure files are available below the reviewer comments at the end of this letter.

If applicable, we recommend that you deposit your laboratory protocols in protocols.io to enhance the reproducibility of your results. Protocols.io assigns your protocol its own identifier (DOI) so that it can be cited independently in the future. For instructions see: https://journals.plos.org/plosone/s/submission-guidelines#loc-laboratory-protocols. Additionally, PLOS ONE offers an option for publishing peer-reviewed Lab Protocol articles, which describe protocols hosted on protocols.io. Read more information on sharing protocols at https://plos.org/protocols?utm_medium=editorial-email&utm_source=authorletters&utm_campaign=protocols.

As the corresponding author, your ORCID iD is verified in the submission system and will appear in the published article. PLOS supports the use of ORCID, and we encourage all coauthors to register for an ORCID iD and use it as well. Please encourage your coauthors to verify their ORCID iD within the submission system before final acceptance, as unverified ORCID iDs will not appear in the published article. *Only*  the individual author can complete the verification step; PLOS staff *cannot*  verify ORCID iDs on behalf of authors.

We look forward to receiving your revised manuscript.

Kind regards,

Aleksandra Klisic

Academic Editor

PLOS One

Journal Requirements:

When submitting your revision, we need you to address these additional requirements.

1. Please ensure that your manuscript meets PLOS ONE's style requirements, including those for file naming. The PLOS ONE style templates can be found at

https://journals.plos.org/plosone/s/file?id=wjVg/PLOSOne_formatting_sample_main_body.pdf and

https://journals.plos.org/plosone/s/file?id=ba62/PLOSOne_formatting_sample_title_authors_affiliations.pdf

2. The ethical approval number(s) listed in the manuscript and/or submission metadata does not match the approval number on the ethical approval document you provided. Please ensure that all approval numbers are correct.

3. Please update your submission to use the PLOS LaTeX template. The template and more information on our requirements for LaTeX submissions can be found at http://journals.plos.org/plosone/s/latex.

4. Thank you for stating the following in the Acknowledgments Section of your manuscript:

“None”

We note that you have provided funding information that is not currently declared in your Funding Statement. However, funding information should not appear in the Acknowledgments section or other areas of your manuscript. We will only publish funding information present in the Funding Statement section of the online submission form.

Please remove any funding-related text from the manuscript and let us know how you would like to update your Funding Statement. Currently, your Funding Statement reads as follows:

“The author(s) received no specific funding for this work.”

Please include your amended statements within your cover letter; we will change the online submission form on your behalf.

5. In the online submission form you indicate that your data is not available for proprietary reasons and have provided a contact point for accessing this data. Please note that your current contact point is a co-author on this manuscript. According to our Data Policy, the contact point must not be an author on the manuscript and must be an institutional contact, ideally not an individual. Please revise your data statement to a non-author institutional point of contact, such as a data access or ethics committee, and send this to us via return email. Please also include contact information for the third party organization, and please include the full citation of where the data can be found.

6. Your ethics statement should only appear in the Methods section of your manuscript. If your ethics statement is written in any section besides the Methods, please delete it from any other section.

7. If the reviewer comments include a recommendation to cite specific previously published works, please review and evaluate these publications to determine whether they are relevant and should be cited. There is no requirement to cite these works unless the editor has indicated otherwise.

[Note: HTML markup is below. Please do not edit.]

Reviewers' comments:

Reviewer's Responses to Questions

**Comments to the Author**

1. Is the manuscript technically sound, and do the data support the conclusions?

The manuscript must describe a technically sound piece of scientific research with data that supports the conclusions. Experiments must have been conducted rigorously, with appropriate controls, replication, and sample sizes. The conclusions must be drawn appropriately based on the data presented.

Reviewer #1: Yes

Reviewer #2: Yes

**********

2. Has the statistical analysis been performed appropriately and rigorously?

Reviewer #1: No

Reviewer #2: Yes

**********

3. Have the authors made all data underlying the findings in their manuscript fully available?

The PLOS Data policy requires authors to make all data underlying the findings described in their manuscript fully available without restriction, with rare exception (please refer to the Data Availability Statement in the manuscript PDF file). The data should be provided as part of the manuscript or its supporting information, or deposited to a public repository. For example, in addition to summary statistics, the data points behind means, medians and variance measures should be available. If there are restrictions on publicly sharing data—e.g. participant privacy or use of data from a third party—those must be specified.

Reviewer #1: No

Reviewer #2: Yes

**********

4. Is the manuscript presented in an intelligible fashion and written in standard English?

PLOS ONE does not copyedit accepted manuscripts, so the language in submitted articles must be clear, correct, and unambiguous. Any typographical or grammatical errors should be corrected at revision, so please note any specific errors here.

Reviewer #1: No

Reviewer #2: Yes

**********

5. Review Comments to the Author

Please use the space provided to explain your answers to the questions above. You may also include additional comments for the author, including concerns about dual publication, research ethics, or publication ethics. (Please upload your review as an attachment if it exceeds 20,000 characters)

Reviewer #1: 1. The primary concern relates to the study design and sampling strategy. Participants were selected using a non-probability consecutive sampling technique from a single ward of Bharatpur Metropolitan City. This approach limits representativeness and introduces selection bias. Since the sample was not randomly selected and came from one localized semi-urban area, the derived NC cutoff values may not generalize to broader pediatric populations in Nepal with different ethnic, nutritional, socioeconomic, or rural–urban backgrounds. The authors should discuss this limitation more explicitly and avoid overgeneralization of conclusions.

2. A second major issue concerns the statistical analysis of overweight prediction. Only 31 children (10.3%) were classified as overweight, yet logistic regression produced a very large adjusted odds ratio (AOR = 20.817, 95% CI: 6.34–68.29). With such a relatively small number of outcome events, regression estimates may be unstable and confidence intervals wide. The manuscript should clarify the number of variables entered into the model, assess events-per-variable adequacy, and explain whether assumptions of logistic regression were fully met. In addition, odds ratios for a continuous predictor such as NC should specify the unit increase (per 1 cm increase or categorized threshold).

3. The ROC analysis is interesting, but the manuscript should provide more detail regarding cutoff derivation and validation. The proposed NC thresholds were developed and tested using the same dataset, which may overestimate performance. No internal validation methods (bootstrapping, split-sample validation, cross-validation) or external validation cohort were used. Therefore, the reported sensitivity, specificity, and AUC values should be interpreted cautiously. Independent validation in another Nepali pediatric cohort would substantially strengthen the manuscript.

4. Another concern is the data availability statement. The manuscript states that data are available from the corresponding author upon reasonable request. PLOS ONE generally expects the minimal underlying dataset to be publicly available unless justified restrictions apply. The authors should deposit de-identified data in a suitable repository or provide a stronger rationale for restricted access. Several potentially important confounders were not adequately addressed. Although age and sex were adjusted in one model, other factors such as pubertal stage, dietary habits, physical activity, parental BMI, socioeconomic status, and ethnicity may influence both NC and overweight status. Because children aged 6–11 years may vary in developmental stage, this should be acknowledged more clearly.

5. Use of BMI-for-age as the Only Reference Standard:

The study evaluates neck circumference against BMI-for-age alone. While BMI is widely used, it does not directly measure body fat distribution or adiposity. Since NC is proposed as an alternative screening marker, comparison with additional standards such as skinfold thickness, waist-to-height ratio, or body composition methods would strengthen validity.

6. Very High Underweight Prevalence (41.3%):

The sample had a strikingly high proportion of underweight children, much higher than overweight cases (10.3%). This unusual nutritional distribution may affect ROC-derived cutoffs and may not represent broader school-age populations. Authors should discuss whether the cutoff values are specific to this malnutrition-burdened population.

7. Cross-Sectional Design Limits Predictive Claim:

The title uses “predictor,” but this is a cross-sectional study. It identifies association and classification ability, not prospective prediction of future overweight. Wording should be revised to avoid implying causal or longitudinal predictive power.

8. Older Statistical Software:

Use of SPSS version 16 is not itself fatal, but it is outdated. Authors should confirm methods were properly implemented and report exact procedures used for ROC and regression analyses.

9. Measurement Reliability Not Reported:

Anthropometric measurements were performed by one investigator, but no intra-observer reliability, repeated measurement error, or calibration consistency data were provided. Since NC differences of less than 1 cm affect cutoffs, measurement precision is important.

Reviewer #2: Thank you for your nice work.

-Neck circumference is labeling more children as normal (68.7) vs BMI based normal range (48.3%).

-NC is labeling more children as overweight (31.3%) vs BMI based overweight (10.3 %). Can you please provide explanation

-On Table 6: BMI has three categories while NC has two. Are the underweight children be classified as normal in the NC group? Please provide explanation and if it has resulted in the above discrepance as well

-Please provide explanation for the relatively wide confidence interval (6.34–68.29)

**********

6. PLOS authors have the option to publish the peer review history of their article (what does this mean?). If published, this will include your full peer review and any attached files.

If you choose “no”, your identity will remain anonymous but your review may still be made public.

**Do you want your identity to be public for this peer review?**  For information about this choice, including consent withdrawal, please see our Privacy Policy.

Reviewer #1: **Yes:**  Shake Ibna Abir

Reviewer #2: **Yes:**  Abraham Sisay Abie

**********

[NOTE: If reviewer comments were submitted as an attachment file, they will be attached to this email and accessible via the submission site. Please log into your account, locate the manuscript record, and check for the action link "View Attachments". If this link does not appear, there are no attachment files.]

To ensure your figures meet our technical requirements, please review our figure guidelines: https://journals.plos.org/plosone/s/figures

You may also use PLOS’s free figure tool, NAAS, to help you prepare publication quality figures: https://journals.plos.org/plosone/s/figures#loc-tools-for-figure-preparation.

NAAS will assess whether your figures meet our technical requirements by comparing each figure against our figure specifications.

# Author comment: Author response to Decision Letter 1

**DOI:** 10.1371/journal.pone.0354997.r002

14 Jun 2026

General Comments

1.Please ensure that your manuscript meets PLOS ONE's style requirements, including those for file naming.

Thank you for this important comment. The manuscript has undergone thorough editing to meet the journal’s style.

Entire manuscript (Pages 1-End)

2.The ethical approval number(s) listed in the manuscript and/or submission metadata does not match the approval number on the ethical approval document you provided. Please ensure that all approval numbers are correct.

Thank you for highlighting this important issue. This has been corrected with the Approval No.: CMC-IRC/081/082-115.

2.4 Ethics statement (Page 7)

3. Please remove any funding-related text from the manuscript and let us know how you would like to update your Funding Statement.

Thank you for this valuable comment. It has been removed from the manuscript. The statement “The authors received no specific funding for this work” has been included in the cover letter.

Declarations (Page 18)

4. In the online submission form you indicate that your data is not available for proprietary reasons and have provided a contact point for accessing this data. Please note that your current contact point is a co-author on this manuscript. According to our Data Policy, the contact point must not be an author on the manuscript and must be an institutional contact, ideally not an individual. Please revise your data statement to a non-author institutional point of contact, such as a data access or ethics committee, and send this to us via return email. Please also include contact information for the third-party organization, and please include the full citation of where the data can be found.

Thank you for this important comment. It has been changed to the data that support the findings of this study are available as supplementary file. We confirm that all underlying data have been made fully available as supplementary files in accordance with the journal requirements.

Supplementary file

5. Your ethics statement should only appear in the Methods section of your manuscript. If your ethics statement is written in any section besides the Methods, please delete it from any other section.

Thank you for this observation. Ethics statement are written in methods section only.

2.4 Ethics statement (Page 7)

6. If the reviewer comments include a recommendation to cite specific previously published works, please review and evaluate these publications to determine whether they are relevant and should be cited. There is no requirement to cite these works unless the editor has indicated otherwise.

Thank you for this valuable comment.

Reviewer 1

1. The primary concern relates to the study design and sampling strategy. Participants were selected using a non-probability consecutive sampling technique from a single ward of Bharatpur Metropolitan City. This approach limits representativeness and introduces selection bias. Since the sample was not randomly selected and came from one localized semi-urban area, the derived NC cutoff values may not generalize to broader pediatric populations in Nepal with different ethnic, nutritional, socioeconomic, or rural–urban backgrounds. The authors should discuss this limitation more explicitly and avoid overgeneralization of conclusions.

Thank you for this insightful comment. Participants were recruited from a single semi-urban site using non-probability sampling, which restricts external validity and may limit the generalizability of these findings to other populations. Although this study proposes locally derived cutoff values of NC, these require further validation. Larger, multicenter studies are warranted to establish standardized NC reference charts, confirm their clinical utility, and yield more precise estimates. These are discussed in the limitation section.

4.4 Limitations (Page-16)

2. A second major issue concerns the statistical analysis of overweight prediction. Only 31 children (10.3%) were classified as overweight, yet logistic regression produced a very large adjusted odds ratio (AOR = 20.817, 95% CI: 6.34–68.29). With such a relatively small number of outcome events, regression estimates may be unstable and confidence intervals wide. The manuscript should clarify the number of variables entered into the model, assess events-per-variable adequacy, and explain whether assumptions of logistic regression were fully met. In addition, odds ratios for a continuous predictor such as NC should specify the unit increase (per 1 cm increase or categorized threshold).

Thank you for this observation. All the predictors within the multivariable logistic regression model satisfied the condition of coloniality through the variance inflation factor (VIF). The OR was calculated adjusted to the variables age and sex. Regarding the model adequacy, the overall goodness of fit is indicated by non-significant chi square value (p>0.05). The Hosmer- Lameshow statistics has chi square value of 2.238 and a p value of 0.97, which means that the p value is not statistically significant. So, this model is quite a good fit. The pseudo R square estimates indicate approximately 12.3% or 25.2% of variation in the dependent variable. The relatively small number of participants in the overweight category may have inflated sensitivity estimates and reduced the precision and generalizability of the diagnostic accuracy measures. It is included in the limitation section.

3. Results (Pages 12-13)

4.4 Limitations (Page-16)

3. The ROC analysis is interesting, but the manuscript should provide more detail regarding cutoff derivation and validation. The proposed NC thresholds were developed and tested using the same dataset, which may overestimate performance. No internal validation methods (bootstrapping, split-sample validation, cross-validation) or external validation cohort were used. Therefore, the reported sensitivity, specificity, and AUC values should be interpreted cautiously. Independent validation in another Nepali pediatric cohort would substantially strengthen the manuscript.

Thank you for this valuable comment. The issue raised are valid and are incorporated in the limitations. Independent validation in another Nepali pediatric cohort would definitely strengthen the findings. This is included in the conclusion.

5. Conclusions (Page- 17)

4. Another concern is the data availability statement. The manuscript states that data are available from the corresponding author upon reasonable request. PLOS ONE generally expects the minimal underlying dataset to be publicly available unless justified restrictions apply. The authors should deposit de-identified data in a suitable repository or provide a stronger rationale for restricted access. Several potentially important confounders were not adequately addressed. Although age and sex were adjusted in one model, other factors such as pubertal stage, dietary habits, physical activity, parental BMI, socioeconomic status, and ethnicity may influence both NC and overweight status. Because children aged 6–11 years may vary in developmental stage, this should be acknowledged more clearly.

Thank you for this important comment. It has been changed to the data that support the findings of this study are available as supplementary file. We confirm that all underlying data have been made fully available as supplementary files in accordance with the journal requirements.

The suggestion has been incorporated in limitations.

Declaration (Page 18)

Supplementary file

4.4 Limitations (Page-16)

5. Use of BMI-for-age as the Only Reference Standard:

The study evaluates neck circumference against BMI-for-age alone. While BMI is widely used, it does not directly measure body fat distribution or adiposity. Since NC is proposed as an alternative screening marker, comparison with additional standards such as skinfold thickness, waist-to-height ratio, or body composition methods would strengthen validity.

Thank you for this important comment. The study indicated a statistically significant positive correlation between neck circumference and height, weight, BMI, waist circumference, and hip circumference (p <0.001 for all). In contrast, no significant correlation was observed between neck circumference and waist-hip ratio (p=0.386).

3. Results Table 8 (Page-12)

6. Very High Underweight Prevalence (41.3%):

The sample had a strikingly high proportion of underweight children, much higher than overweight cases (10.3%). This unusual nutritional distribution may affect ROC-derived cutoffs and may not represent broader school-age populations. Authors should discuss whether the cutoff values are specific to this malnutrition-burdened population.

Thank you for this important comment. The cutoff values are calculated specific to the overweight children. 3. Results (Page 9-11)

7. Cross-Sectional Design Limits Predictive Claim:

The title uses “predictor,” but this is a cross-sectional study. It identifies association and classification ability, not prospective prediction of future overweight. Wording should be revised to avoid implying causal or longitudinal predictive power.

Thank you for this valuable comment. The title of the study has been revised to Neck circumference as an anthropometric marker of overweight among school-aged children in Bharatpur, Nepal: a community-based study.

(Page 1)

8. Older Statistical Software:

Use of SPSS version 16 is not itself fatal, but it is outdated. Authors should confirm methods were properly implemented and report exact procedures used for ROC and regression analyses.

Thank you for this valuable comment. SPSS version 16 is freely available for use. New versions though available are not free and the use of corrupted version seemed unethical so were not used. The authors confirm that the analysis methods were implemented and reported properly.

9.Measurement Reliability Not Reported:

Anthropometric measurements were performed by one investigator, but no intra-observer reliability, repeated measurement error, or calibration consistency data were provided. Since NC differences of less than 1 cm affect cutoffs, measurement precision is important.

Thank you for this valuable comment. All measurements were taken twice and the average of two readings was used for the analysis. To maintain intra observer reliability same standardized protocol, same anatomical landmark, same position, and the same measuring tape was used each time for all the measurements.

2.2 Data collection (Pages 6-7)

Reviewer #2

1Thank you for your nice work.

-Neck circumference is labeling more children as normal (68.7) vs BMI based normal range (48.3%).

-NC is labeling more children as overweight (31.3%) vs BMI based overweight (10.3 %). Can you please provide explanation

Thank you for this valuable comment. The NC has been recategorized as overweight and not overweight. Regarding the higher percentage of overweight children using NC, it solely depends on single parameter i.e. neck circumference. It does not consider the height and weight of the children. Larger studies are required to confirm the association. This is included in limitation

3. Results (Page 12)

4.4 Limitations (Page-16)

2. On Table 6: BMI has three categories while NC has two. Are the underweight children be classified as normal in the NC group? Please provide explanation and if it has resulted in the above discrepancy as well

Thank you for this valuable comment. The underweight children were classified as normal in the NC group previously which has been corrected. The NC has been recategorized as overweight and not overweight. 3. Results (Page 12)

3.Please provide explanation for the relatively wide confidence interval (6.34–68.29)

Confidence interval was very wide, reflecting low precision and considerable uncertainty regarding the true magnitude of the effect. Larger, multicenter studies are warranted to establish standardized NC reference charts, confirm their clinical utility, and yield more precise estimates.

4.4 Limitations (Page-16)

Attachment Submitted filename: Response to Reviewers.docx

# Peer review: Decision Letter 1

**DOI:** 10.1371/journal.pone.0354997.r003

**Authors:** Aleksandra Klisic

16 Jul 2026

Neck circumference as an anthropometric marker of overweight among school-aged children in Bharatpur, Nepal: a community-based study

PONE-D-26-16450R1

Dear Dr. Bajracharya,

We’re pleased to inform you that your manuscript has been judged scientifically suitable for publication and will be formally accepted for publication once it meets all outstanding technical requirements.

Within one week, you’ll receive an e-mail detailing the required amendments. When these have been addressed, you’ll receive a formal acceptance letter and your manuscript will be scheduled for publication.

An invoice will be generated when your article is formally accepted. Please note, if your institution has a publishing partnership with PLOS and your article meets the relevant criteria, all or part of your publication costs will be covered. Please make sure your user information is up-to-date by logging into Editorial Manager at Editorial Manager® and clicking the ‘Update My Information' link at the top of the page. For questions related to billing, please contact billing support.

If your institution or institutions have a press office, please notify them about your upcoming paper to help maximize its impact. If they’ll be preparing press materials, please inform our press team as soon as possible -- no later than 48 hours after receiving the formal acceptance. Your manuscript will remain under strict press embargo until 2 pm Eastern Time on the date of publication. For more information, please contact onepress@plos.org.

Kind regards,

Aleksandra Klisic

Academic Editor

PLOS One

Additional Editor Comments (optional):

Reviewers' comments:

Reviewer's Responses to Questions

**Comments to the Author**

1. If the authors have adequately addressed your comments raised in a previous round of review and you feel that this manuscript is now acceptable for publication, you may indicate that here to bypass the “Comments to the Author” section, enter your conflict of interest statement in the “Confidential to Editor” section, and submit your "Accept" recommendation.

Reviewer #2: All comments have been addressed

**********

2. Is the manuscript technically sound, and do the data support the conclusions?

The manuscript must describe a technically sound piece of scientific research with data that supports the conclusions. Experiments must have been conducted rigorously, with appropriate controls, replication, and sample sizes. The conclusions must be drawn appropriately based on the data presented.

Reviewer #2: Yes

**********

3. Has the statistical analysis been performed appropriately and rigorously?

Reviewer #2: Yes

**********

4. Have the authors made all data underlying the findings in their manuscript fully available?

The PLOS Data policy requires authors to make all data underlying the findings described in their manuscript fully available without restriction, with rare exception (please refer to the Data Availability Statement in the manuscript PDF file). The data should be provided as part of the manuscript or its supporting information, or deposited to a public repository. For example, in addition to summary statistics, the data points behind means, medians and variance measures should be available. If there are restrictions on publicly sharing data—e.g. participant privacy or use of data from a third party—those must be specified.

Reviewer #2: No

**********

5. Is the manuscript presented in an intelligible fashion and written in standard English?

PLOS ONE does not copyedit accepted manuscripts, so the language in submitted articles must be clear, correct, and unambiguous. Any typographical or grammatical errors should be corrected at revision, so please note any specific errors here.

Reviewer #2: Yes

**********

6. Review Comments to the Author

Please use the space provided to explain your answers to the questions above. You may also include additional comments for the author, including concerns about dual publication, research ethics, or publication ethics. (Please upload your review as an attachment if it exceeds 20,000 characters)

Reviewer #2: (No Response)

**********

7. PLOS authors have the option to publish the peer review history of their article (what does this mean?). If published, this will include your full peer review and any attached files.

If you choose “no”, your identity will remain anonymous but your review may still be made public.

**Do you want your identity to be public for this peer review?**  For information about this choice, including consent withdrawal, please see our Privacy Policy.

Reviewer #2: **Yes:**  Abraham Sisay Abie

**********

# Editor report: Acceptance letter

**DOI:** 10.1371/journal.pone.0354997.r004

**Authors:** Aleksandra Klisic

PONE-D-26-16450R1

PLOS One

Dear Dr. Bajracharya,

I'm pleased to inform you that your manuscript has been deemed suitable for publication in PLOS One. Congratulations! Your manuscript is now being handed over to our production team.

At this stage, our production department will prepare your paper for publication. This includes ensuring the following:

* All references, tables, and figures are properly cited

* All relevant supporting information is included in the manuscript submission,

* There are no issues that prevent the paper from being properly typeset

You will receive further instructions from the production team, including instructions on how to review your proof when it is ready. Please keep in mind that we are working through a large volume of accepted articles, so please give us a few days to review your paper and let you know the next and final steps.

Lastly, if your institution or institutions have a press office, please let them know about your upcoming paper now to help maximize its impact. If they'll be preparing press materials, please inform our press team within the next 48 hours. Your manuscript will remain under strict press embargo until 2 pm Eastern Time on the date of publication. For more information, please contact onepress@plos.org.

You will receive an invoice from PLOS for your publication fee after your manuscript has reached the completed accept phase. If you receive an email requesting payment before acceptance or for any other service, this may be a phishing scheme. Learn how to identify phishing emails and protect your accounts at https://explore.plos.org/phishing.

If we can help with anything else, please email us at customercare@plos.org.

Thank you for submitting your work to PLOS ONE and supporting open access.

Kind regards,

PLOS ONE Editorial Office Staff

on behalf of

Dr. Aleksandra Klisic

Academic Editor

PLOS One
